# Initialization of Nanowire or Cluster Growth Critically Controlled by the Effective V/III Ratio at the Early Nucleation Stage

**DOI:** 10.1021/acs.jpclett.3c00484

**Published:** 2023-05-04

**Authors:** Chen Chen, Yanmeng Chu, Linjun Zhang, Haojun Lin, Wenzhang Fang, Zheyu Zhang, Chaofei Zha, Kejia Wang, Hui Yang, Xuezhe Yu, James A. Gott, Martin Aagesen, Zhiyuan Cheng, Suguo Huo, Huiyun Liu, Ana M. Sanchez, Yunyan Zhang

**Affiliations:** † College of Information Science and Electronic Engineering, Zhejiang University, Hangzhou 310027, China; ‡ School of Micro-Nano Electronics, Zhejiang University, Hangzhou, Zhejiang 311200, China; § College of Photonic and Electronic Engineering, Fujian Normal University, Fuzhou 350117, Fujian, China; ∥ Institute for Materials Discovery, University College London, Roberts Building, Malet Place, London WC1E 7JE, United Kingdom; ⊥ Department of Electronic and Electrical Engineering, University College London, London WC1E 7JE, United Kingdom; # Department of Physics, University of Warwick, Coventry CV4 7AL, United Kingdom; @ Center for Quantum Devices, Niels Bohr Institute, University of Copenhagen, Universitetsparken 5, 2100 Copenhagen, Denmark; ∇ London Centre for Nanotechnology, University College London, London WC1H 0AH, United Kingdom

## Abstract

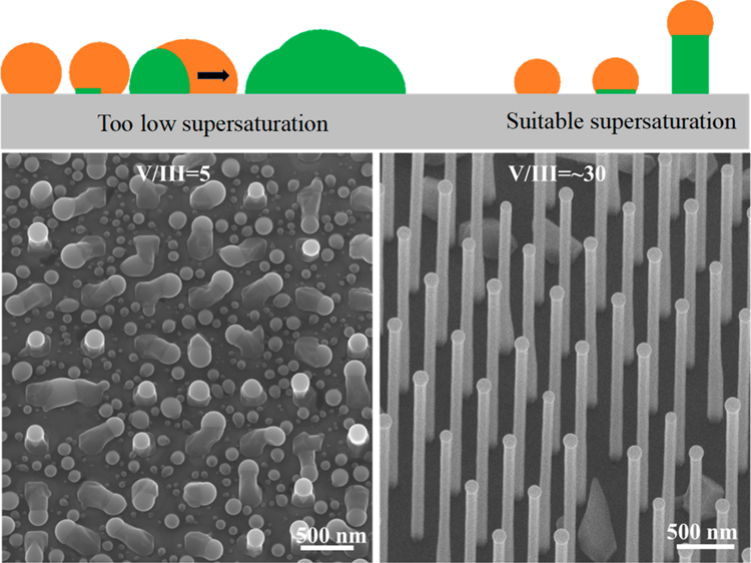

For self-catalyzed nanowires (NWs), reports on how the catalytic droplet initiates successful NW growth are still lacking, making it difficult to control the yield and often accompanying a high density of clusters. Here, we have performed a systematic study on this issue, which reveals that the effective V/III ratio at the initial growth stage is a critical factor that governs the NW growth yield. To initiate NW growth, the ratio should be high enough to allow the nucleation to extend to the entire contact area between the droplet and substrate, which can elevate the droplet off of the substrate, but it should not be too high in order to keep the droplet. This study also reveals that the cluster growth between NWs is also initiated from large droplets. This study provides a new angle from the growth condition to explain the cluster formation mechanism, which can guide high-yield NW growth.

Nanowires (NWs) with a quasi-one-dimensional morphology have many potential novel optoelectronic and microelectronic applications, in devices such as light emitters, photovoltaics, and high-speed electronics.^1−7^ Their strong ability to be integrated into silicon (Si) substrates allows great flexibility in device design and the potential for low-cost fabrication,^8,9^ as well as seamless integration with the Si industrial platform, solving the III–V/Si integration challenge that has existed for more than 40 years.^10,11^

Self-catalyzed NW growth is one of the most popular growth modes, which has the advantage of COMS compatibility.^12−16^ However, this mode is highly complicated, especially the nucleation process at the beginning growth stage. Many studies have tried to explain the NW growth mechanism,^17−20^ such as the nucleation sites in patterned substrate growth.^21^ Nevertheless, a majority of these studies were performed in gold-catalyzed growth mode, which is quite different from self-catalyzed mode.^22−24^ In self-catalyzed growth, the catalytic droplet consists of a group III metal that is significantly different from gold, with a lower surface energy.^25^ In addition, the non-consumable feature of Au in the droplets makes them have quite different growth window ranges, such as the V/III flux ratio, growth temperature, etc.^26,27^ Thus, it was found that the Au-catalyzed mode cannot be directly used for the self-catalyzed mode.^28^ Therefore, the factors that control the initiation of successful self-catalyzed NW growth are still unclear. In addition, unoptimized growth tends to produce a high density of clusters alongside the low-yield NWs when the growth is on unpatterned substrates, and quite often on patterned substrates.^29−31^ The clusters are nano/micro-sized lump/mound structures on the substrate surface, which are the parasitic materials deposited during NW growth. Their growth consumes a large amount of materials that should have otherwise been contributed to NW growth, and their presence can cover a large surface area of the substrate and reduce the space for NWs. Moreover, cluster enlargement can embrace the NWs and cut down the material supply for NW growth, which can cause the shrinkage of the catalytic droplet size and hence NW diameter, leading to the generation of defects (commonly stacking faults) in NWs^24^ and even the termination of growth. The clusters themselves normally contain a high density of defects, which can cause carrier loss by nonradiative recombination.^32^ In the case of tandem devices, these clusters can cause severe current leakage and decrease in breakdown voltages. For tandem solar cells,^33,34^ the clusters can also reduce the device efficiency by consuming and/or reflecting photons and blocking and/or reducing the penetration of light into the bottom cells.

To the best of our knowledge, detailed reports on how the droplet initiates successful NW growth at the beginning growth stage are still lacking, due to the lack of suitable tools and/or methods for investigating this stage. Although the change in the surface energy of the droplet can lead to the failure of NW growth and promotion of cluster growth, it commonly happens when using extra elements (such as Be) with a high solubility inside the catalytic droplet.^23^ However, in most cases, the growth without introducing any extra element can still lead to the formation of clusters. Therefore, cluster formation is widely attributed to the nature of the substrate surface, such as the chemical composition, thermodynamic stability, and wetting properties of the surface oxide controlled by its thickness.^35−38^ These factors were shown to determine the catalytic droplet volume and curvature, and the configurations within the pinhole, which will lead to either NW or cluster growth. However, NW growth is a highly complicated process, and the nature of the substrate surface is only one of the important factors. Careful optimization may change the unfavorable growth condition into favorable for NW growth. Therefore, it is necessary to gain more insights into the nucleation mechanism during the initial growth stage and uncover the link between the growth condition and NW/cluster growth, through which growth can maximize the NW yield and suppress cluster formation. This type of study, however, remains lacking.

In this study, the nucleation mechanism of the self-catalyzed NW growth at the initial growth stage is investigated using Ga-catalyzed NWs, which reveals that the effective V/III ratio at this stage plays a critical role in controlling the nucleation types that can lead to the growth of either NWs or clusters.

During the very initial growth stage (with a duration of only 3 min), the sample surface is covered by two types of discrete droplets (Figure 1a). A majority of the droplets are ∼80 nm in diameter and crouching on the substrate surface in the saucer shape (left inset of Figure 1a). Nucleation can be seen at some parts of the edge. Here, we call these droplets “type I” droplets. The other type of droplet (right insets of Figure 1a) is ball-shaped and much smaller (∼50 nm). They stand vertically on the substrate. The percentage of this type of droplet is very small, as one can see that there is only one in Figure 1a. Here, we call these droplets “type II” droplets. With an increase in the growth duration to 8 min (Figure 1b), the type I droplets are still crouching on the substrate but seem to be pushed by a long solid tail and crawling forward. The tail is wedge-shaped and is larger at the droplet end, suggesting that the droplet size was increasing during growth. We call them “crawling NWs”. Many more type II droplets appear on the sample. With an increase in the growth duration to 13 min (Figure 1c), the type I droplets are still crawling on the substrate. Their tail is much longer, and quite a high percentage of them are still in a wedge shape shown in the left inset of Figure 1c. The type II droplets are clearly off of the substrate with a short segment of NW beneath (right inset of Figure 1c). After long duration growth for 60 min, the NWs are quite long (∼3 μm). A majority of them are standing vertically on the substrate with the diameter uniform along the length (Figure 1d). On top of each NW is a round droplet, showing clear Ga-catalyzed growth. The sample surface has high-density large clusters alongside the NWs that are normally full of defects as one can see in the transmission electron microscopy (TEM) images in panels a and b of Figure 2. It must be emphasized that there is no observation of direct cluster growth without a droplet from Figure 1a–c.

**Figure 1:**
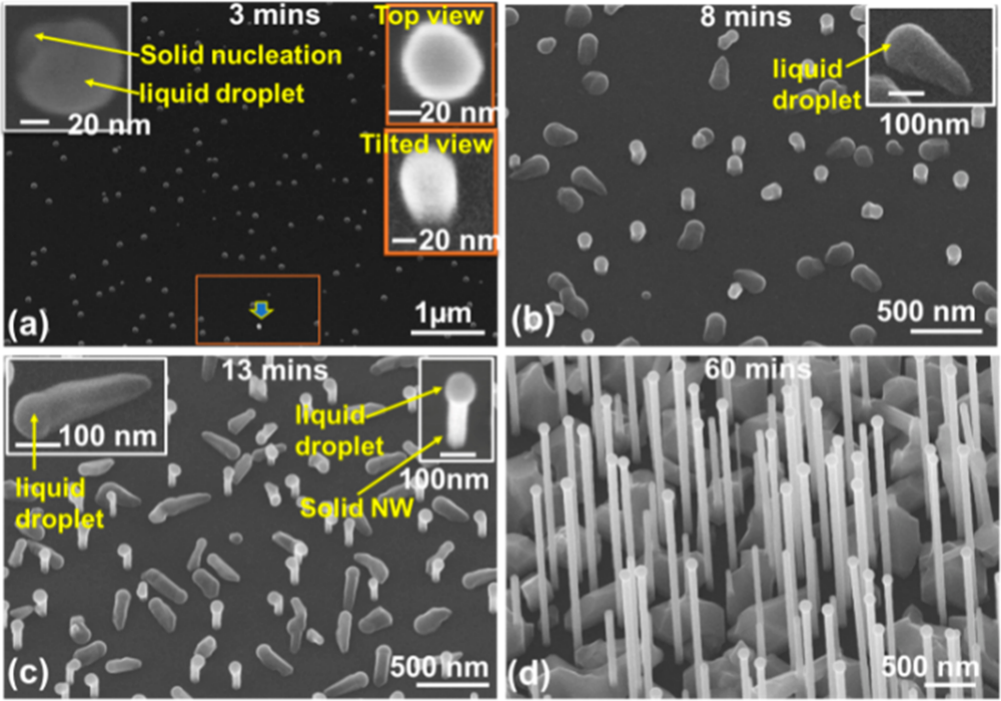
Scanning electron microscopy (SEM) images of GaAs NWs grown at 630 °C and a V/III flux ratio of 50 with different durations: (a) 3, (b) 8, (c) 13, and (d) 60 min. In panel d, the NW direction is slightly off of vertical due to the nonflat sample mounting during the SEM measurement.

**Figure 2:**
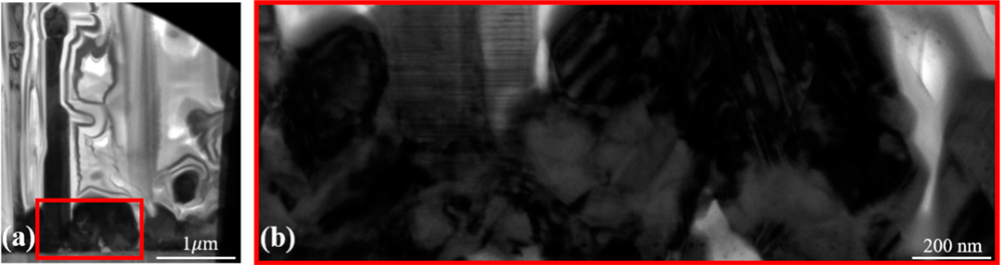
(a) Low- and (b) high-magnification TEM images of the GaAs(P) clusters enclosed in the red square in panel a.

According to the phase diagram of the As–Ga system, the solubility of group V elements inside the group III droplet is very low (<0.7%) at ∼700 °C, which makes the droplet supersaturation level sensitive to the V/III ratio.^39^ The V/III flux ratio is thus one of the most important factors that can strongly influence NW growth.^40^ Thus, GaAs NWs were grown with different flux ratios (*F*_As_/*F*_Ga_). As shown in Figure 3a, when the sample is grown with a low flux ratio of 25, the majority of the sample surface is covered by interlinked clusters, leaving only a few separated small areas available for NW growth. There are only a few NWs with a very short length (∼400 nm) and a quite large diameter (180 nm). One round droplet can be seen on top of each NW. With an increase in the flux ratio to 40 (Figure 3b), the cluster area shrinks significantly, making them more separate. There are more NWs growing, and the NW length is much longer (∼2 μm) with a tapered shape. With a further increase in the flux ratio to 50 (Figure 3c), the cluster shrinks to small islands, and the majority of the surface area is exposed for NW growth. The NWs are quite long (∼3 μm). A majority of the NWs are standing vertically on the substrate, with a few exceptions in different directions.^41^ Their density is much higher than in panels a and b of Figure 3, and their diameter is uniform along the length. Some NWs protrude from the top of the clusters (see the red arrow in Figure 3c), as they are embraced by cluster enlargement during growth. This type of NW is in general shorter and does not have a droplet on the top, which suggests that the cluster cuts off the Ga supply and causes the droplet to be consumed during NW growth. This phenomenon can also be seen in panels a and b of Figure 3 but is more difficult to observe due to the short NW length. Finally, the flux ratio is increased to 120. This sample (Figure 3d) is fully covered by dense clusters. There are only a few nanopillars that have very short lengths (100–600 nm) and a large diameter (∼200 nm).

**Figure 3:**
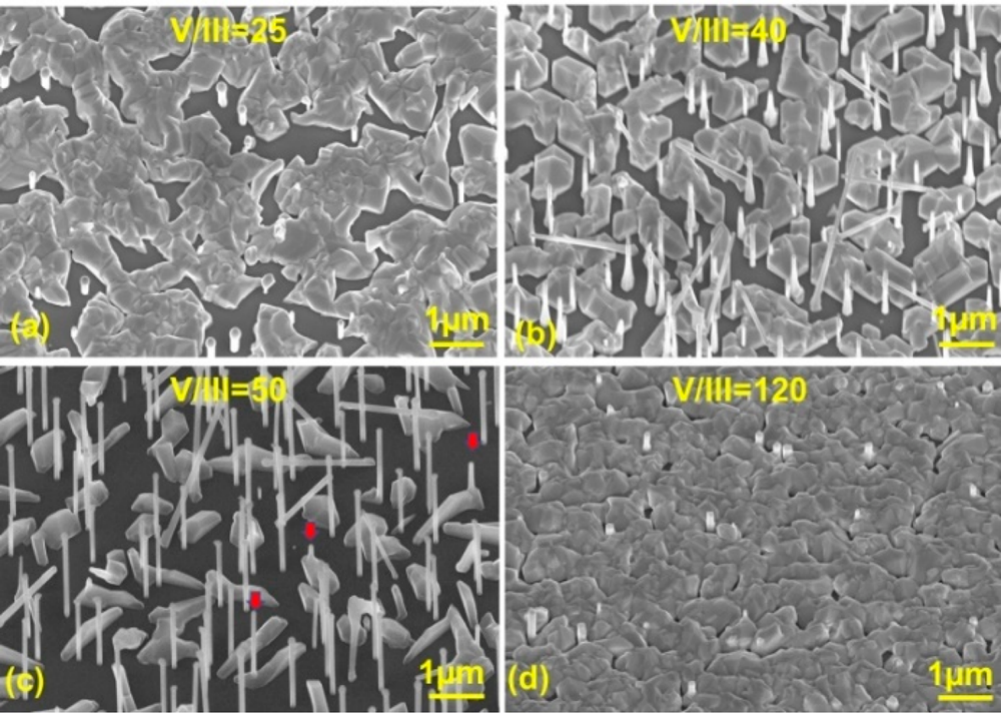
SEM images of GaAs NWs grown at the optimized growth temperature of ∼630 °C for 60 min with different V/III flux ratios: (a) 25, (b) 40, (c) 50, and (d) 120.

This suggests that it is very difficult to form droplets at an overly high flux ratio. Even if there was droplet formation, they can only survive for a very short time to produce very short NWs. Afterward, the shell growth on the NW stumps makes them thicker. It is quite straightforward to understand cluster formation with overly high flux ratios as it can hinder the formation of catalytic droplets;^42^ it is interesting to see the increased level of cluster formation with a decrease in the flux ratio.

The growth temperature can also strongly affect NW and cluster growth. Therefore, GaAs NWs are grown at different temperatures. As shown in Figure 4a, when grown at a high temperature of ∼640 °C, the sample surface is dominated by clusters with an elongated shuttle shape that is sharper at both ends. Only a few NWs are growing but with an extremely low density. With a ∼5 °C decrease in temperature (Figure 4b), more NWs are showing up but with very short lengths, similar to Figure 1c. With a further decrease in the temperature to ∼630 °C (Figure 4c), the cluster density is reduced significantly and the NW density and length are increased greatly, similar to Figure 1d.

**Figure 4:**
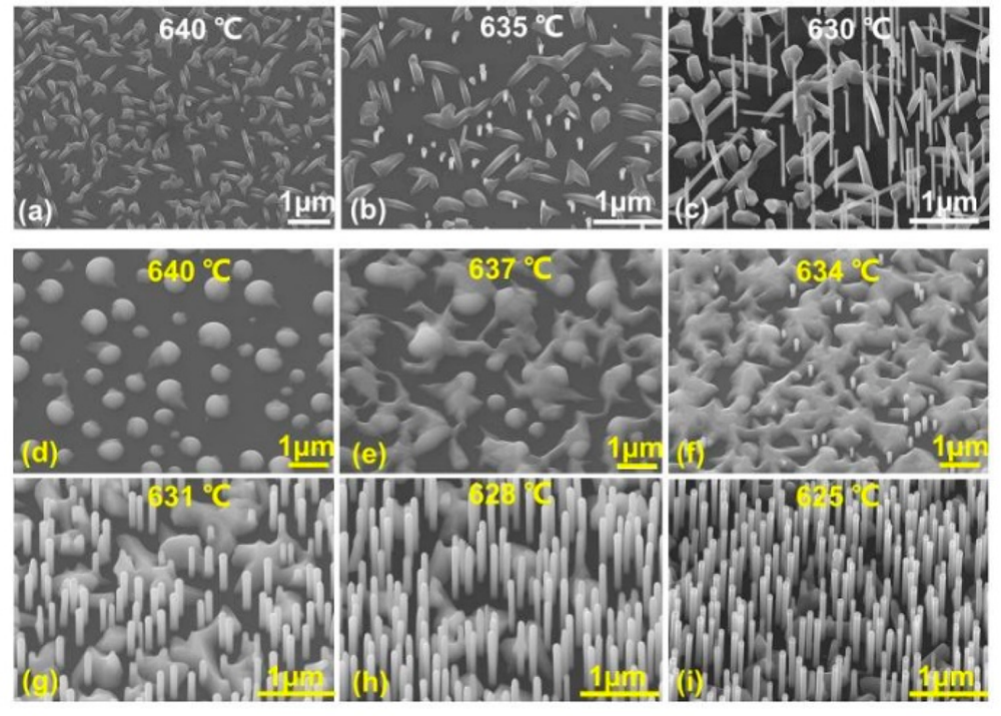
SEM images of (a–c) GaAs NWs and (d–i) GaAsSb NWs grown at different temperatures.

To gain more insight into the correlation between the growth temperature and NW growth, GaAsSb NWs are also grown at different temperatures. The chemical activity of Sb is much lower than that of As, which can greatly slow the nucleation process. As shown in Figure 4d, when grown at a temperature of ∼640 °C, the sample surface is distributed with separate droplets that are partially solidified. With a decrease in temperature to ∼637 °C (Figure 4e), the droplets are linked with each other by the solidified crystal bridges, suggesting a faster nucleation rate. With a further decrease in temperature to ∼634 °C (Figure 4f), the droplets are solidified into clusters and cover a majority of the area of the sample surface, indicating greatly enhanced nucleation ability. Meanwhile, there are a few droplet-catalyzed NWs growing in the small spaces between the clusters. With an even further decrease in temperature to 631–625 °C (Figure 4g–i), the cluster coverage shrinks significantly while the NW density and length increase dramatically.

From the observation presented above, it can be deduced that the clusters and NWs originate from the type I and type II droplets, respectively, if the growth with an overly high V/III flux ratio is not taken into consideration (Figure 3d). In addition, Figure 1 shows that the type I droplets are grown earlier than the type II droplets, which means that only when the type I droplet develops to a certain level are the type II droplets allowed to initiate. An effective V/III ratio-controlled or supersaturation-controlled nucleation mechanism is proposed in Figure 5a.

**Figure 5:**
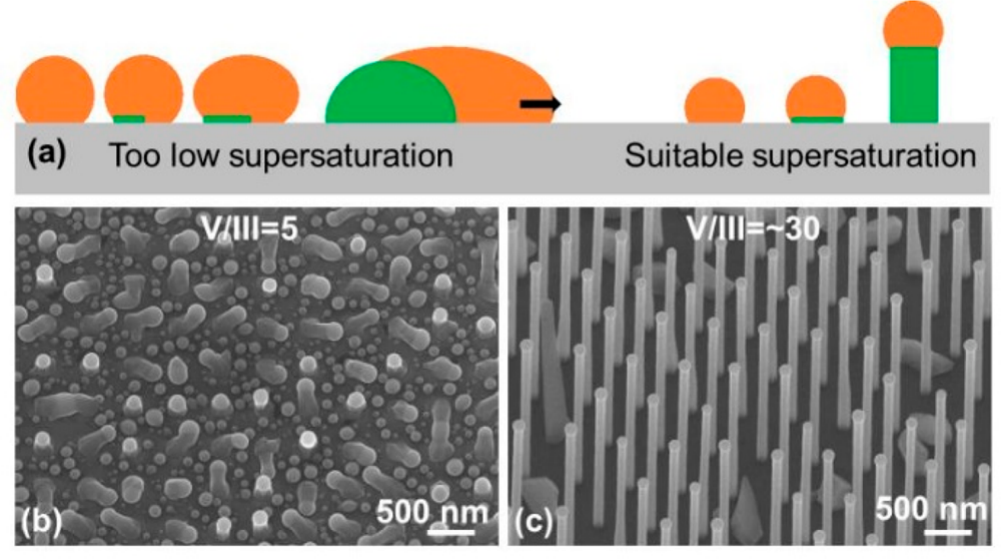
(a) Illustration of the nucleation mechanism for clusters and NWs. SEM images of GaAsP NWs grown on patterned Si substrates with V/III flux ratios during growth of (b) 5 and (c) ∼30.

The self-catalyzed growth mode needs a catalytic nanodroplet to assist the growth that consists of group III metals, such as Ga or In, forming a group III element-rich environment, and NW growth normally occurs at a high temperature (around the eutectic temperature).^43^ When the growth starts with fluxes open, the metal droplet forms. The droplet bottom is normally connected to the substrate lattice through the existing pinhole openings or the ones etched by themselves. During NW growth, the introduction of group V flux can increase the degree of supersaturation of the droplet above a certain threshold. Then, the nucleation of one new atomic layer begins at the bottom of the Ga droplet and grows across the bottom of the droplet area. The growth of the atomic layer at the same time decreases the level of supersaturation inside the droplet, and it takes a while (depending on the effective V/III ratio and other factors) before the level of supersaturation increases again to allow the next layer growth.

If the growth occurs with a low V/III flux ratio, supersaturation refilling inside the droplet will be slow, and hence, the nucleation will be slow. In addition, during the initial growth stage, the nucleation area (pinholes) may be small and/or the droplet may also need some time to etch through the oxide into the substrate,^44^ which can further slow the nucleation. In this case, the nucleation rate cannot match the metal material supply/collection speed, and the droplet size can increase very quickly. As a result, nucleation can only partly cover the bottom area of the droplet, which is shown in Figure 1a. Panels d and e of Figure 4 also show similar phenomena, which is because the group V element evaporates more efficiently than the group III ones at the high growth temperature, causing the low level of supersaturation inside the droplet. Because the nucleation develops from one side, the droplet is pushed forward and crawls along the same direction with the nucleation, leaving a crystal tail behind and becoming crawling NWs (Figures 1b,c and 4d,e). As the droplet enlargement is much faster than the nucleation, the crawling NWs develop into a wedge shape (insets of panels b and c of Figure 1). It must be mentioned that there is no influence of native oxide in our patterned growth, as one can see in the Experimental Section.

The surface of the crawling NWs can also provide a lattice template for nucleation, and its enlargement/elongation can thus create a larger nucleation area, hastening the consumption of the group III material. Therefore, the growth environment gradually changes from being rich in group III to being rich in normal/suitable group III. The decrease in the supply of group III material and the enhanced nucleation rate gradually cause the droplet of the crawling NWs to shrink and disappear eventually. This should be why the clusters in panels a and b of Figure 4 show a shuttle shape that is sharp at both ends. After the droplet is consumed, the vapor–solid shell growth happens on the surface of the clusters, making them larger. As the majority of the Ga for NW growth diffuses from the substrate, especially during the initial stage when the NW is very short, the cluster enlargement can also reduce the supply of group III material to the droplet of vertical NWs, which can cause the droplet to shrink and develop into a tapered shape in Figure 3b.

Alongside the development of clusters, more new droplets can form. These newly formed droplets are commonly smaller due to the reduced supply of group III material, which is in accord with panels a and b of Figure 1. The non-uniform droplet size distribution in Figure 4d might also be due to this reason. At the same time, the effective V/III ratio (defined as the group III and V fluxes that are not consumed by the clusters and can directly contribute to NW growth) on the sample surface continues to increase. At a certain point, the effective V/III ratio is high enough to form nucleation layers to fully cover the bottom area of these newly formed small droplets, which can elevate them off of the substrate. These droplets are then developed into type II droplets mentioned before and start the off-substrate NW growth using the layer-by-layer growth mode.^45−48^ Therefore, the clusters formed during the initial growth stage can tune the effective V/III flux ratio and make the environment suitable for vertical NW growth. The increase in the III/V ratio and the decrease in the growth temperature can also have a similar effect to increase the effective V/III ratio inside the droplet and significantly increase the NW yield, which can be seen in Figures 3 and 4.

According to this theory, the achievement of high-yield NW and suppression of clusters requires a well-balanced effective V/III flux ratio and carefully controlled droplet size, which can be achieved by using the patterned substrates that can accurately control the interwire pitch and hence the source material collection area.^42,46^ Although electron beam lithography (EBL) is widely used in research, a cost-effective method will be nanoimprint lithography (NIL) which can be widely accepted by the market.^42^ In addition, we have developed a high-temperature in situ pattern cleaning technology to guarantee a clean surface in the patterned holes and thus can provide a lattice template for NW growth.^42^ As shown in Figure 5b, when the NW grows with a low V/III flux ratio of 5 on patterned substrates, there is one large cluster crawling out of each patterned hole with a droplet on the tip. The surface of the SiO_2_ pattern has a high density of Ga droplets, which clearly indicates the environment is quite rich in group III. Thus, the V/III flux ratio was increased to ∼30 in the second growth. As shown in Figure 5c, the NW with a high yield was successfully grown, and there is no Ga droplet between the NWs, suggesting a well-balanced V/III flux ratio. There are some clusters among the NWs, which could be due to the poor quality of the patterned substrate that is made by nanoimprint lithography. There have many reports of much higher yields with the use of high-quality substrates made of electron beam lithography.^49,50^

In summary, the nucleation mechanism at the initial stage for guiding the self-catalyzed growth toward either the NW or the cluster has been studied systematically using Ga-based NWs. The cluster grown between NWs was also found to be initialized from the droplet-catalyzed mode, indicating a low V/III flux ratio. The low level of supersaturation inside the droplet can only support the regional nucleation happen at the bottom of the droplet, which leads to the growth of crawling NWs. The enlargement of the crawling NWs can provide a larger nucleation area to enhance the rate of consumption of group III material, which causes droplet shrinkage and changes them into clusters in the end. With the clusters consuming excessive group III materials, smaller droplets can form successively, and the effective V/III ratio on the substrate is also increased. The nucleation layer can thus fully cover the bottom of these small droplets and elevate them off of the substrate for successful vertical NW growth. Therefore, the clusters formed during the initial growth stage can tune the effective V/III flux ratio and make the environment suitable for vertical NW growth. To achieve high-yield NW growth, patterned substrates are recommended, as it can well balance the effective V/III flux ratio and carefully control the droplet size. This study provides insight into the nucleation mechanism during the initial NW growth stage, which can guide the growth of high-yield NWs and the suppression of cluster formation. It will hence promote the development of high-performance devices, especially those with tandem structures.

## Experimental Section

*NW Growth*. All of the NWs used here were Ga-catalyzed and grown on unpatterned or patterned Si substrates.^40,42^ The GaAs and GaAsP nanowires were grown directly on Si substrates by solid-source III–V molecular beam epitaxy (MBE). If not mentioned specifically, GaAs NWs were grown with a Ga beam equivalent pressure, a V/III flux ratio, a substrate temperature, and a growth duration of 8.41 × 10^–8^ Torr, 50, ∼630 °C, and 1 h, respectively. GaAsSb NWs were grown with a Ga beam equivalent pressure, a V/III flux ratio, a P/(As + P) flux ratio, and a growth duration of 8.41 × 10^–8^ Torr, 44, 20%, and 1 h, respectively. For GaAsP NW growth on patterned substrates, the growth started with a high-temperature deoxidization step to clean the patterned holes, and then growth was performed with a Ga flux of 1.6 × 10^–7^ Torr, V/III flux ratios between 5 and 30, a P/(P + As) flux ratio of 12%, and a temperature of ∼630 °C throughout the 45 min growth duration. The substrate temperature was measured by a pyrometer. For each type of substrate, the preparation procedures were kept the same to guarantee almost identical surface conditions, and the details can be found in refs (42) and (51).

*Scanning Electron Microscopy (SEM)*. The time-dependent evolution of NWs and clusters was studied first with GaAs NWs grown with different durations and characterized by SEM. The NW morphology was measured with a Zeiss XB 1540 FIB/SEM system.

*Transmission Electron Microscopy (TEM)*. The TEM measurements were performed on JEOL 2100 and doubly corrected ARM200F microscopes, both operating at 200 kV.

## Supporting Information Available

Transparent Peer Review report available (PDF)
