# Supplementary material for: Initialization of Nanowire or Cluster Growth Critically Controlled by the Effective V/III Ratio at the Early Nucleation Stage
Source: J Phys Chem Lett. 2023 May 4;14(19):4433–9. doi: 10.1021/acs.jpclett.3c00484 (PMC10202363; doi:10.1021/acs.jpclett.3c00484)
Supplement: Supplementary file 1 — jz3c00484_si_001.pdf [file jz3c00484_si_001.pdf]

jz-2023-00484j.R1

Name: Peer Review Information for "The Initialization of Nanowire or Cluster Growth Critically Controlled by the Effective V/III Ratio at the Early Nucleation Stage"

First Round of Reviewer Comments

Reviewer: 1

Comments to the Author

Integrating III-Vs onto silicon platform is the holy grail of the semiconductor field for decades, which is however still a big challenge. The nanowire structure provides a highly promising solution to it. Self-catalyzed growth has the advantages of fast growth, contamination free, high crystal quality, which is a popular research topic. However, this growth mechanism is novel and need further exploration. The nucleation mechanism is one key factor determining the nanowire growth, but still no one knows how to control it to achieve high-yield nanowire growth. This work provide an insight into this topic and explain the nucleation at early stage of growth with quite in detailed experiments which show the tuning to the parameters. Thus, this is a valuable research and is at the right level to be published in The Journal of Physical Chemistry Letters.

To further improve the manuscript, I suggest the following minor revisions:

1) It would be great for the authors to show the cluster details with such as TEM.

2) The patterned substrate is a good method to suppress the growth of clusters. But it is too expensive to use. How to make this method being accepted by market?

3) There have been some works focus on the nucleation mechanism of gold-catalyzed mode. But this mode is quite different form self-catalyzed mode. So, there nucleation mechanism is very different as well. Authors should explain clearly this in the manuscript.

4) There are some typos in the manuscript. The authors should check it again.

Reviewer: 2

Comments to the Author

The authors staged a parametric investigation on the self-catalyzed growth of GaAs, GaAsSb, GaAsP nanowires (NWs), which have been heavily studied in previous works in the literature. It was claimed that slim and high density of NWs can only be grown with high droplet supersaturation of Group V elements within Group III catalyst droplets during the initial nucleation stage. I have no problem with this systematic fundamental work, which is valuable, but have the follow suggestions for further improvements.

1) All the experimental observations could also be explained from the view point of size control of the Ga catalyst droplet, not necessarily the supersaturation. For example, Fig. 1 indicates only the fact that formation of small droplet of Ga is necessary for an easy kicking off growth in vertical direction, while apparently the large ones are difficult to be lift up. In Fig. 2, the reduction of Ga supply is favorable for the formation of tiny droplets for vertical growth, while too high flow V/III ratio totally suppresses the formation of surplus Ga droplet, undermining the NW growth.

2) Of course, I agree that a higher concentration of Group V element in Ga droplet will be easier to establish in smaller droplets, but this is not always equivalent to a high supersaturation. Actually, high supersaturation is good for initiating the nucleation overcoming the initial interfacial energy barrier, but in the following expansion growth of the nucleus or nuclei, it is the absolute concentration of Group V element that determines the growth rate, that is how fast a monolayer can be completed at the bottom of the droplet for pushing vertical growth.

3) Again, the emerging of slim NW growth upon large granules on ground also reflects that the formation of tiny Ga droplet is easier on uneven and sharp wedge lines, which is not the case for the initiate stage on flat surface.

4) I would not agree that the claim that: “Self-catalyzed NW growth … has the advantages of fast growth rate, COMS compatible, good crystal quality”. How fast is the self-catalyzed growth and compared to what reference? I guess it is CMOS compatible, right? But usually, NWs are supposed to be integrated as horizontal channels for electronics, so any idea to do that with the self-catalyzed growth? For light emission or harvesting it is OK, but has nothing to do with CMOS.

5) The substrate and patterning procedure should be specified and explained, and why an etching is needed in “…the nucleation area (pinholes) maybe small and/or the droplet may also need some time to etch through the oxide into the substrate” for the benefit of the readers.

Author's Response to Peer Review Comments:

Re: Manuscript ID: jz-2023-00484j
Title: The Initialization of Nanowire or Cluster Growth Critically Controlled by the Supersaturation at the Early Nucleation Stage

Dear Prof. Editor and reviewers,

Thank you so much for getting this manuscript reviewed. We sincerely appreciate the helpful feedback and valuable comments from the reviewers, and also the opportunity for us to improve the manuscript. We have made the required revision to the manuscript which we believe have addressed all the comments brought out by the reviewers, which indeed strengthens the paper considerably. The responses to the reviewers and main revisions made to the manuscript are listed below:

Manuscript Formatting Request from the Editorial office:

Comment 1:

Abstract: Shorten the abstract to 150 words or fewer. (Currently 207.)
Response 1:

The Abstract has been shortened to exactly 150 words.

Comment 2:

Headers: Remove the section heading(s) throughout the body of the manuscript (you can leave Methods and Abstract headings).
Response 2:

The section headings throughout the body of the manuscript have been removed.

Comment 3:

*References: Please fix the style of all references to use JPCL formatting (check all references carefully). ***JPC Letters reference formatting requires that journal references should contain: () around numbers, author names, article title (titles entirely in title case or entirely in lower case), abbreviated journal title (italicized), year (bolded), volume (italicized), and pages (first-last). Book references should contain author names, book title (in the same pattern), publisher, city, and year. Websites must include date of access.*
Response 3:

Thanks a lot for pointing out these. All the references have been updated with JPCL formatting.

Comment 4:

Please submit your publication files without any markups. Any copies that contain highlights, colored text, or tracked changes should be submitted as "Supporting Information for Review Only."
Response 4:

Thank you for your reminder. Two copies of files have been added into the system: one without any change trackers are added as the “manuscript”; while the other with change markers are *submitted as "Supporting Information for Review Only."*

Comments from Reviewer: 1

Integrating III-Vs onto silicon platform is the holy grail of the semiconductor field for decades, which is however still a big challenge. The nanowire structure provides a highly promising solution to it. Self-catalyzed growth has the advantages of fast growth, contamination free, high crystal quality, which is a popular research topic. However, this growth mechanism is novel and need further exploration. The nucleation mechanism is

one key factor determining the nanowire growth, but still no one knows how to control it to achieve high-yield nanowire growth. This work provides an insight into this topic and explain the nucleation at early stage of growth with quite in detailed experiments which show the tuning to the parameters. Thus, this is a valuable research and is at the right level to be published in The Journal of Physical Chemistry Letters.

***Recommendation**: This paper is publishable subject to minor revisions noted. Further review is not needed.*

Comment 1:

It would be great for the authors to show the cluster details with such as TEM.

Response 1:

Thanks a lot for this suggestion.

It is indeed great to add in the TEM images, which has been done in new version. We also add the instruction of the pictures: "The sample surface has high-density large clusters alongside the NWs which are normally full of defects as can be seen by a presentative transmission electron microscope (TEM) images shown in Fig. 2a~b."

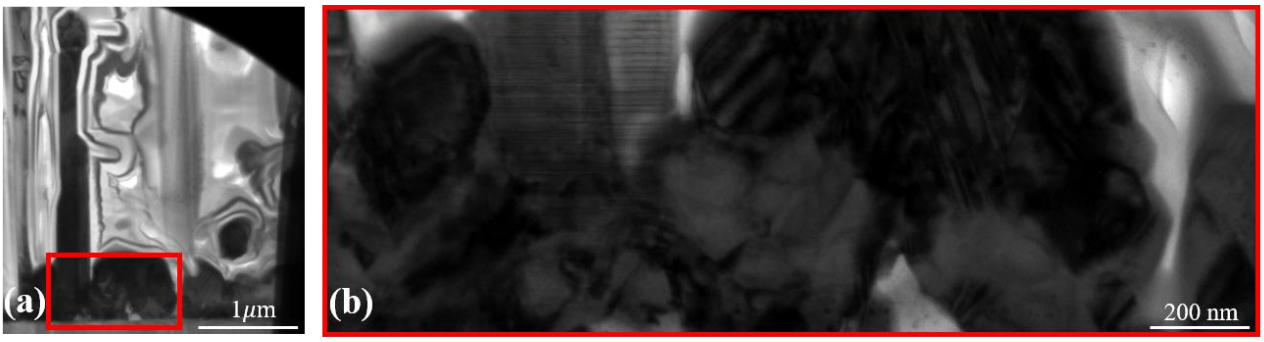

Fig. 2. (a) low- and (b) high-magnification TEM image of the GaAs(P) clusters enclosed in the red square in part (a).

Comment 2:

The patterned substrate is a good method to suppress the growth of clusters. But it is too expensive to use. How to make this method being accepted by market?

Response 2:

Thanks a lot for pointing out this.

For practical use, the nanoimprint lithography (NIL) is a highly cost-effective technique that is capable of made wafer-scale patterns with high throughput. There are already many reports on NIL-positioned III−V NWs (*Munshi, A. M. et al. Nano Lett 2014, 14 (2), 960–966. Zhang, Y. et al. Nano Lett 2014, 14 (8), 4542–4547.) This has been added in the revised manuscript*:

"Although the electron beam lithography (EBL) is widely used in the research, a cost-effective method will be nanoimprint lithography (NIL) that can be widely accepted by the market[42,46].

(42) Zhang, Y.; Wu, J.; Aagesen, M.; Holm, J.; Hatch, S.; Tang, M.; Huo, S.; Liu, H. Self-Catalyzed Ternary Core-Shell GaAsP Nanowire Arrays Grown on Patterned Si Substrates by Molecular Beam Epitaxy. *Nano Lett* **2014**, *14* (8), 4542–4547. https://doi.org/10.1021/nl501565b.

(46) Munshi, A. M.; Dheeraj, D. L.; Fauske, V. T.; Kim, D. C.; Huh, J.; Reinertsen, J. F.; Ahtapodov, L.; Lee, K. D.; Heidari, B.; Van Helvoort, A. T. J.; Fimland, B. O.; Weman, H. Position-Controlled Uniform GaAs Nanowires on Silicon Using Nanoimprint Lithography. *Nano Lett* **2014**, *14* (2), 960–966. https://doi.org/10.1021/nl404376m."

Comment 3:

There have been some works focus on the nucleation mechanism of gold-catalyzed mode. But this mode is quite different form self-catalyzed mode. So, there nucleation mechanism is very different as well. Authors should explain clearly this in the manuscript.

Response 3:

Thanks a lot for this good suggestion.

The following description has been added into the manuscript to give readers a more complete knowledge background:

"In the self-catalyzed growth, the catalytic droplet is made of group-III metal which has significant difference from the gold, such as the lower surface energy[23]. Besides, the non-consumable feature of Au in the droplets makes them have quite different growth window range, such as V/III flux ratio, growth temperature et al[24,25]. Thus, the discoveries found from the Au-catalyzed mode cannot be directly used for self-catalyzed mode[26]."

(23) Zhang, Y.; Sanchez, A. M.; Aagesen, M.; Fonseka, H. A.; Huo, S.; Liu, H. Droplet Manipulation and Horizontal Growth of High-Quality Self-Catalysed GaAsP Nanowires. *Nano Today* **2020**, *34*, 100921. https://doi.org/10.1016/j.nantod.2020.100921.

(24) Gil, E.; Dubrovskii, V. G.; Avit, G.; André, Y.; Leroux, C.; Lekhal, K.; Grecenkov, J.; Trassoudaine, A.; Castelluci, D.; Monier, G.; Ramdani, R. M.; Robert-Goumet, C.; Bideux, L.; Harmand, J. C.; Glas, F. Record Pure Zincblende Phase in GaAs Nanowires down to 5 Nm in Radius. *Nano Lett* **2014**, *14* (7), 3938–3944. https://doi.org/10.1021/nl501239h.

(25) Dheeraj, D. L.; Munshi, A. M.; Scheffler, M.; van Helvoort, A. T. J.; Weman, H.; Fimland, B. O. Controlling Crystal Phases in GaAs Nanowires Grown by Au-Assisted Molecular Beam Epitaxy. *Nanotechnology* **2013**, *24* (1), 015601. https://doi.org/10.1088/0957-4484/24/1/015601.

(26) Joyce, H. J.; Gao, Q.; Tan, H. H.; Jagadish, C.; Kim, Y.; Zhang, X.; Guo, Y.; Zou, J. Twin-Free Uniform Epitaxial GaAs Nanowires Grown by a Two-Temperature Process. *Nano Lett* **2007**, *7* (4), 921–926. https://doi.org/10.1021/nl062755v.

Comment 4:

There are some typos in the manuscript. The authors should check it again.

Response 4:

We sincerely appreciate the careful reading of the reviewer. We have gone through the manuscript carefully for the typos check.

Comments from Reviewer: 2

I have no problem with this systematic fundamental work, which is valuable, but have the follow suggestions for further improvements.

Recommendation*: This paper may be publishable, but major revision is needed; I would like to be invited to review any future revision.*

Comment 1:

All the experimental observations could also be explained from the view point of size control of the Ga catalyst droplet, not necessarily the supersaturation. For example, Fig. 1 indicates only the fact that formation of small droplet of Ga is necessary for an easy kicking off growth in vertical direction, while apparently the large ones are difficult to be lift up. In Fig. 2, the reduction of Ga supply is favorable for the formation of tiny droplets for vertical growth, while too high flow V/III ratio totally suppresses the formation of surplus Ga droplet, undermining the NW growth.

Of course, I agree that a higher concentration of Group V element in Ga droplets will be easier to establish in smaller droplets, but this is not always equivalent to a high supersaturation. Actually, high supersaturation is good for initiating the nucleation and overcoming the initial interfacial energy barrier, but in the following expansion growth of the nucleus or nuclei, it is the absolute concentration of Group V element that determines the growth rate, that is how fast a monolayer can be completed at the bottom of the droplet for pushing vertical growth.

Response 1:

Actually, I think we are talking on the same explanation. When there are no clusters on the sample surface, the Ga collection area is large and effective V/III is low. The large supply of the Ga lead to the formation of large droplets. When the cluster formed to consume a large portion of Ga, the effective V/III getting higher with the enlargement of the clusters. This can lead to the formation of small droplet.

We do agree with this reviewer that the "*supersaturation*" can be misleading. Thus, it is replaced by "effective V/III ratio", which is clearer to readers.

Comment 2:

Again, the emerging of slim NW growth upon large granules on ground also reflects that the formation of tiny Ga droplet is easier on uneven and sharp wedge lines, which is not the case for the initiate stage on flat surface.
Response 2:

Yes, this could be possible. However, in this research, we are focusing on the influence of the change of growth condition. From the Fig. 5, we used the patterned substrates, which can show clearly this point, as the patterned holes does not allow the droplet have free location choice. So, we can eliminate this variable.

Comment 3:

I would not agree that the claim that: "Self-catalyzed NW growth ... has the advantages of fast growth rate, COMS compatible, good crystal quality". How fast is the self-catalyzed growth and compared to what reference? I guess it is CMOS compatible, right? But usually, NWs are supposed to be integrated as horizontal channels for electronics, so any idea to do that with the self-catalyzed growth? For light emission or harvesting it is OK, but has nothing to do with CMOS.
Response 3:

Thanks a lot for pointing out this.

It is indeed difficult to compare the growth rate, as it is strongly growth condition controlled. It is too careless to jump to this statement. So, we deleted this according to the suggestion of this reviewer.
For "*CMOS compatible*", this type of NWs can be used for GAA-FET, or make Si photonic devices. Actually, one of the main aims of the researchers to develop the "self-catalyzed" NWs is for not using Au that is not "*CMOS compatible*".

Comment 4:

The substrate and patterning procedure should be specified and explained, and why an etching is needed in "…the nucleation area (pinholes) maybe small and/or the droplet may also need some time to etch through the oxide into the substrate" for the benefit of the readers.
Response 4:

Thank you for your kind suggestion.
"…the nucleation area (pinholes) maybe small and/or the droplet may also need some time to etch through the oxide into the substrate", which is for the growth on un-patterned substrates. The substrate surface is normally covered by a layer of native oxide but with some small pinholes. The droplet needs to tech through the oxide layer or make the pinhole bigger for the sake of growth.
For the patterned substrate, this step is un-necessary, as there is a high-temperature de-oxidization step to clean the patterned holes. "*Zhang, Y. et al. Nano Lett 2014, 14 (8), 4542–4547.*".
To better explain this, we add it into the experimental section, and also the following description in the manuscript:
"It needs to be mentioned that there is no influence from native oxide in our patterned growth, as can be seen from the experimental section."
"For GaAsP NW growths on patterned substrates, the growth started with a high-temperature de-oxidization step to clean the patterned holes,….."

Prof. Yunyan Zhang
School of Micro-Nano Electronics, Zhejiang University,
Hangzhou, Zhejiang, 311200, China
&
DEPARTMENT OF ELECTRONIC & ELECTRICAL ENGINEERING
LONDON CENTRE FOR NANOTECHNOLOGY
University College London, Torrington Place, WC1E 7JE
&
Universität Paderborn
NW/P P8.2.11, Warburger St. 100, 33098 Paderborn, Germany
Tel.: +44 (0)7543285907

jz-2023-00484j.R2

Name: Peer Review Information for "The Initialization of Nanowire or Cluster Growth Critically Controlled by the Effective V/III Ratio at the Early Nucleation Stage"

Second Round of Reviewer Comments

Reviewer: 2

Comments to the Author

The authors have addressed most of my comments satisfactorily in their response and the revised manuscript. I think it is now ready for publication in JPCL, except one point that:

They stated, in response to my 3rd comment, that "For “CMOS compatible”, this type of NWs can be used for GAA-FET, or make Si photonic devices."

-- The use of this vertical self-catalyzed NWs for GAA-FET, in CMOS compatible way, sounds amazing, but actually impossible at all. Could the authors provide any example for the use of catalytic nanowires for GAA channels? Indeed, what is needed for GAA-FET device includes a key capability to stack several layers of horizontal nanowires.

Reviewer: 1

Comments to the Author

The authors have addressed all my questions

Author's Response to Peer Review Comments:

Re: Manuscript ID: jz-2023-00484j
Title: The Initialization of Nanowire or Cluster Growth Critically Controlled by the Supersaturation at the Early Nucleation Stage

Dear Prof. Editor and reviewers,

Thank you so much for getting this manuscript reviewed. We sincerely appreciate the helpful feedback and valuable comments from the reviewers, and also the opportunity for us to improve the manuscript. We have made the required revision to the manuscript which we believe have addressed all the comments brought out by the reviewers, which indeed strengthens the paper considerably. The responses to the reviewers and main revisions made to the manuscript are listed below:

Manuscript Formatting Request from the Editorial office:

Comment 1:

Please label as TOC GRAPHIC without the "S" on the end.

Response 1:

The label as TOC GRAPHIC has been updated without “S”.

Comment 2:

Figure 1. Please include (a-c) in the figure caption.

Figure 3. Please include (a-d) in the figure caption.

Response 2:

Thanks a lot for pointing out these. The figure captions of the manuscript have been updated with letters.

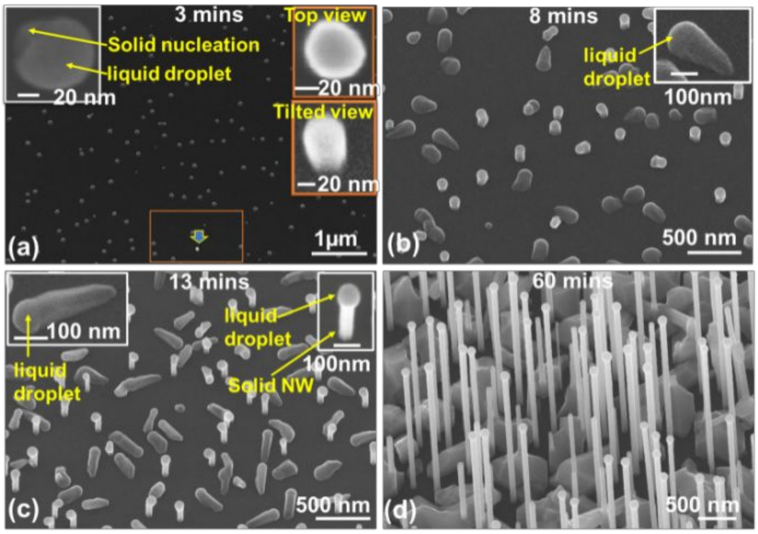

Fig. 1. Scanning electron microscope (SEM) images of GaAs NWs grown at a temperature of 630 ˚C and a V/III flux ratio of 50 with different durations: (a) 3 mins, (b) 8 mins, (c) 13 mins, (d) 60 mins.

In (d), the NW direction slightly off from the vertical direction is due to the un-flat sample mounting during for the SEM measurement.

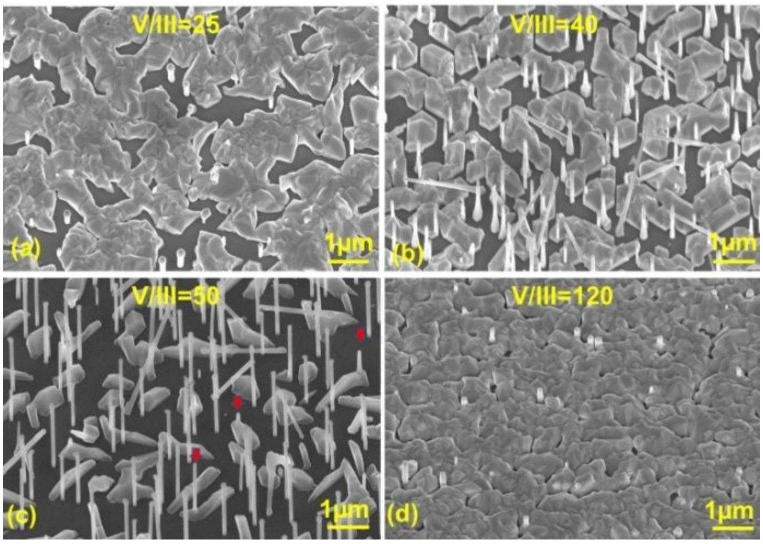

Fig. 3. SEM images of GaAs NWs grown at the optimized growth temperature of ~630 ˚C for 60 mins with different V/III flux ratios: (a) V/III=25, (b) V/III=40, (c) V/III=50, (d) V/III=120.

Comment 3:

Remove the section heading(s) throughout the manuscript (but leave "Abstract" and "TOC Graphic"); i.e., Experimental Section.

Response 3:

Thanks a lot for pointing out this. The "Experimental Section" heading has been removed.

Comment 4:

Please list funding under the Acknowledgement section.

Response 4:

The funding is listed as follows:

The authors acknowledge the support of Leverhulme Trust, EPSRC (grant nos. EP/P000916/1 and EP/P000886/1), and EPSRC National Epitaxy Facility.

Comment 5:

Reference 11 - article title (titles entirely in title case or entirely in lower case). Please correct.

Response 5:

Thanks a lot for pointing out this. This reference has been updated with JPCL formatting

Comments from Reviewer 1

Comment:

They stated, in response to my 3rd comment, that "For "CMOS compatible", this type of NWs can be used for GAA-FET, or make Si photonic devices."

-- The use of this vertical self-catalyzed NWs for GAA-FET, in CMOS compatible way, sounds amazing, but actually impossible at all. Could the authors provide any example for the use of catalytic nanowires for GAA channels? Indeed, what is needed for GAA-FET device includes a key capability to stack several layers of horizontal nanowires.

Response 1:

Thanks a lot for pointing out this. For "*CMOS compatible*", there are already many reports on vertical NWs used for GAA-FET transistors (*Liu, M. et al. Vertical GeSn nanowire MOSFETs for CMOS beyond silicon. Commun Eng 2, 7 (2023). R. Ritzenthaler. et al. 2018 IEEE International Electron Devices Meeting (IEDM). 2018; pp 21.5.1-21.5.4.).* This has been added in the revised manuscript references:

(3) Zhu, Z.; Svensson, J.; Jönsson, A.; Wernersson, L.-E. Performance Enhancement of GaSb Vertical Nanowire P-Type MOSFETs on Si by Rapid Thermal Annealing. *Nanotechnology* **2022**, *33* (7), 075202. https://doi.org/10.1088/1361-6528/ac3689.

(4) Ram, M. S.; Persson, K.-M.; Borg, M.; Wernersson, L.-E. Low-Power Resistive Memory Integrated on III–V Vertical Nanowire MOSFETs on Silicon. *IEEE Electron Device Letters* **2020**, *41* (9), 1432–1435. https://doi.org/10.1109/LED.2020.3013674.

Prof. Yunyan Zhang
School of Micro-Nano Electronics, Zhejiang University,
Hangzhou, Zhejiang, 311200, China
&
DEPARTMENT OF ELECTRONIC & ELECTRICAL ENGINEERING
LONDON CENTRE FOR NANOTECHNOLOGY
University College London, Torrington Place, WC1E 7JE
&
Universität Paderborn
NW/P P8.2.11, Warburger St. 100, 33098 Paderborn, Germany
Tel.: +44 (0)7543285907
